# Marked Reduction of AKT1 Expression and Deregulation of AKT1-Associated Pathways in Peripheral Blood Mononuclear Cells of Schizophrenia Patients

**DOI:** 10.1371/journal.pone.0032618

**Published:** 2012-02-29

**Authors:** Nico J. M. van Beveren, Gabrielle H. S. Buitendijk, Sigrid Swagemakers, Lianne C. Krab, Christian Röder, Lieuwe de Haan, Peter van der Spek, Ype Elgersma

**Affiliations:** 1 Department of Psychiatry, Erasmus University Medical Center, Rotterdam, The Netherlands; 2 Department of Neuroscience, Erasmus University Medical Center, Rotterdam, The Netherlands; 3 Department of Bioinformatics/Department of Genetics, Erasmus University Medical Center, Rotterdam, The Netherlands; 4 Department of Psychiatry, Academic Medical Center, Amsterdam, The Netherlands; 5 Afdeling Nieuwe Kennis, Delta Center for Mental Health Care, Rotterdam, The Netherlands; University of Illinois at Chicago, United States of America

## Abstract

**Background:**

Recent studies have suggested that deregulated AKT1 signaling is associated with schizophrenia. We hypothesized that if this is indeed the case, we should observe both decreased AKT1 expression as well as deregulation of AKT1 regulated pathways in Peripheral Blood Mononuclear Cells (PBMCs) of schizophrenia patients.

**Objectives:**

To examine PBMC expression levels of AKT1 in schizophrenia patients versus controls, and to examine whether functional biological processes in which AKT1 plays an important role are deregulated in schizophrenia patients.

**Methods/Results:**

A case-control study, investigating whole-genome PBMC gene expression in male, recent onset (<5 years) schizophrenia patients (N = 41) as compared to controls (N = 29). Genes, differentially expressed between patients and controls were identified using ANOVA with Benjamini-Hochberg correction (false discovery rate (FDR) = 0.05). Functional aspects of the deregulated set of genes were investigated with the Ingenuity Pathway Analysis (IPA) Software Tool. We found significantly decreased PBMC expression of AKT1 (p<0.001, t = −4.25) in the patients. AKT1 expression was decreased in antipsychotic-free or -naive patients (N = 11), in florid psychotic (N = 20) and in remitted (N = 21) patients. A total of 1224 genes were differentially expressed between patients and controls (FDR = 0.05). Functional analysis of the entire deregulated gene set indicated deregulated canonical pathways involved in a large number of cellular processes: immune system, cell adhesion and neuronal guidance, neurotrophins and (neural) growth factors, oxidative stress and glucose metabolism, and apoptosis and cell-cycle regulation. Many of these processes are associated with AKT1.

**Conclusions:**

We show significantly decreased PBMC gene expression of AKT1 in male, recent-onset schizophrenia patients. Our observations suggest that decreased PBMC AKT1 expression is a stable trait in recent onset, male schizophrenia patients. We identified several AKT related cellular processes which are potentially affected in these patients, a majority of which play a prominent role in current schizophrenia hypotheses.

## Introduction

Whole-genome gene expression studies using peripheral blood cells, have yielded an extensive list of deregulated genes in schizophrenia patients [1]–[5]. Although there are obvious limitations to using these cells to investigate a CNS disease, they do circumvent some of the confounding factors present in post-mortem brain samples of schizophrenia patients, (e.g. life-long exposure to psychotropic medication and drugs of abuse, and the impact of long-term hospitalization). Moreover, lymphocytes can be collected in the early phases of the disorder. Peripheral gene expression can be informative about gene expression in the central nervous system as whole blood shows significant gene expression similarities with multiple central nervous system tissues, and the expression levels of many classes of biologically relevant processes are not significantly different between whole blood and prefrontal cortex [6], [7]. In patients with schizophrenia, a comparison of gene expression profiles from brain tissue with profiles from peripheral blood cells, identified genes that were common to both tissues, confirming the validity of gene expression profiling of blood for detecting schizophrenia biomarkers [3], [4]. Specifically, the combination of gene expression data and bioinformatics provides an opportunity to investigate perturbations in functional processes over alterations in individual gene expression [8].

Converging evidence from association studies has resulted in several candidate risk-genes for schizophrenia (i.e. see Carter [9]). Among these is AKT1, a member of the serine-threonine protein kinase AKT gene family which includes three members (AKT1, AKT2, and AKT3), that possess partially redundant functions and contribute to several cellular functions including cell growth, survival, and metabolism.

Gain or loss of AKT activity has been associated with several human diseases, including cancer and type 2 diabetes [10], [11]. An influential article by Emamian et al. [12] showed a 68% reduction of AKT1 protein levels in lymphocyte-derived cell lines of schizophrenia patients, and presented several additional converging lines of evidence suggesting involvement of AKT1 in schizophrenia, including disturbed prepulse inhibition in AKT−/− mice, decreased expression of AKT1 in post-mortem brains of schizophrenia patients, and association of AKT1 variants with schizophrenia.

Based on the findings of Emamian et al. [12], we hypothesized that AKT1 expression might wel be decreased in PBMCs of schizophrenia patients as compared to controls (*hypothesis 1*). Furthermore we reasoned that reduced AKT1 expression in patients should lead to deregulation of biological processes in which AKT1 plays an important role (*hypothesis 2*). In addition, we reasoned that if reduction of AKT1 was a signature of the disease (either cause or consequence) rather than a response to antipsychotics, it should also be deregulated in antipsychotic-free or -naive patients (*hypothesis 3*).

Since all these hypotheses are dependent on finding different AKT1 levels in PBMCs of patients, we first performed a pilot study in Peripheral Blood Mononuclear Cells (PBMCs) to get a feeling of the required power to proof or disproof this hypothesis. Surprisingly, despite the small study (N = 8 patients vs N = 8 controls) we already observed a significant decreased AKT1 expression in medicated schizophrenia patients (pilot data are summarized in **supporting table S1**). This formed the impetus to perform a larger case-control study, investigating whole-genome PBMC gene expression in schizophrenia patients as compared to controls.

Schizophrenia is considered to be a syndrome with heterogeneous biological underpinnings. One such factor contributing to heterogeneity are gender differences. Male patients with schizophrenia have earlier onset, show more severe symptoms, leading to more frequent hospital admission [13]. It has also been suggested that estrogen activity may play a role in these observed male/female differences. These phenomena may have impact on AKT1 activity. We therefore restricted inclusion to male patients only.

## Methods

### Subject selection

Male patients presenting with recent-onset schizophrenia at the department of psychiatry of the Erasmus University Medical Center, Rotterdam (EMC) were candidate to participate in the study. The EMC operates an early recognition and treatment clinic for patients with psychotic symptoms. Patients are referred to the EMC by primary caretakers and general mental health care facilities throughout the Rotterdam region (1.2 million inhabitants), an industrialized area with a mixed population of Dutch ancestry and immigrants, mainly from Northern-Africa, Turkey and the Dutch West-Indies. Patients are referred to the EMC, with acute psychotic symptoms. The EMC also offers prolonged ambulatory treatment for patients with schizophrenia after remission of acutely psychotic symptoms.

Eligible for inclusion were male patients diagnosed with schizophrenia or schizophreniform disorder according to DSM IV criteria after a Comprehensive Assessment of Symptoms and History interview (CASH) [14] and by consensus between two senior psychiatrists who were blind to the expression results at the time of diagnosis. Additional criteria were recent onset (defined as duration of illness <5 yr) and age >15 and <36 years. Clinical symptom severity was assessed with the Positive And Negative Syndrome Scale (PANNS).

Exclusion criteria were the presence of any somatic or neurological disorders as investigated by routine clinical and laboratory examination performed at admission, and abuse of heroin, cocaine, or alcohol. Cannabis abuse was not an exclusion criterion, to warrant the generalizability of our results, as patients often use cannabis. Concomitant use of mood-stabilizers and/or antidepressants was an exclusion criterion, as these agents are known to influence AKT expression and/or phosphorylation status.

We included stabilized patients from our outpatient clinic as well as acutely psychotic recent-onset patients from our inpatient clinic. In this way we would be able to assess the impact of variable Duration of Illness (DUI) on AKT1 expression, and to assess the effect of prolonged exposure to antipsychotic medication on AKT1 expression. Moreover, inclusion of acutely psychotic, never- or minimally treated patients enabled us to include antipsychotic-naïve and –free patients (see also description of the deferred consent procedure).

Age- matched controls were recruited from the students and staff of the EMC medical school and hospital. The exclusion criteria for the controls were the same as for the patients. Additionally, the presence of psychiatric disorders in first-degree relatives of the controls was also an exclusion criterion. Controls were evaluated for the self-reported presence of somatic, psychiatric or neurological disorders (either in the controls or in their first-degree relatives), medication use, or the presence of drug abuse. Cannabis abuse was, similar to the patients, not an exclusion criterion.

All participating subjects were diagnosed and screened by a single senior psychiatrist (NJMvB) who is trained in the CASH interview, and final inclusion was reached based on consensus between NJMvB and another senior psychiatrist (CR).

Inclusion criteria for the initial pilot study were similar to the ones outlined in the previous paragraph; patients and controls participating in the main study did not participate in the pilot study.

### Consent and deferred consent

All participating subjects provided written informed consent after complete description of the study.

For those patients who were severely psychotic and too disturbed to provide consent, a separate procedure was applied in which informed consent was initially given by a first-degree relative and final written consent was sought within six week from the patients themselves (‘deferred consent’). Blood samples for these patients were collected with a vacutainer system together with the samples which are part of the regular clinical screening, so no additional venipuncture was needed. Blood samples and data from patients who withheld consent after six weeks were destroyed. This procedure enabled us to include also the most severely psychotic patients.

This study was approved (including the deferred consent procedure) by the Erasmus University Medical Center Institutional Review Board and was conducted according to the declaration of Helsinki.

### Sample preparation

We paid meticulous attention to the processing of the blood samples, isolation of RNA and preparation of the microarray in order to minimize methodological variability. Samples from both non-fasting patients and controls were taken around the same time of the day (10–11 am), in the same room, with venipuncture performed by the same (experienced) research nurse. From each participant 30 ml of blood was drawn into heparinized tubes.

All blood samples were non-fasting and obtained between 10.00 and 121.00 am to minimize diurnal variation. Samples were transported immediately to the laboratory facilities, which are within walking distance from the clinical department. All subsequent laboratory procedures, including biotinylation and hybridization to the microarrays were performed by the same analyst.

PBMCs were isolated by Ficoll gradient separation, started within 20 minutes after the drawing of blood and performed with minimum time variation. Cells were subsequently disrupted (Qiashredder kit; Qiagen), and RNA was isolated (RNEasy minikit; Qiagen) with an additional DNAse digestion step (RNAse-free DNAse set; Qiagen),all according to the manufacturer's protocol, diluted in nuclease-free water, and frozen at −80 C before use. Before freezing, RNA purity and quantity was assessed with the NanoDrop ND-1000 spectrophotometer (Nanodrop technologies). After thawing, the isolated RNA was biotinylated into cRNA using the One-Cycle Target Labelling and Control Reagents Kit (Affymetrix Co) according to the manufacturer's protocol. Before hybridization RNA quality and integrity was assessed using the Agilent 2100 BioAnalyzer (Agilent).

Biotinylated cRNA was hybridized to the Affymetrix Human Genome U133 plus 2.0 GeneChip© microarray containing 54,675 probe sets (Affymetrix Co). Each sample was individually biotinylated and hybridized to an individual microarray. Biotinylation was performed in batches with patients and controls both being present in each of the batches. The arrays were scanned and analyzed using Affymetrix Microarray Suite 4.2 software. Scanning was performed in three batches. As samples from 43 patients and 29 controls were available for biotinylation and scanning, patients and controls were present in an approximately 4∶3 ratio in each batch.

All data is MIAME compliant; the raw data has been deposited in the GEO database under accession number GSE27383, scheduled for release July 1^st^, 2013.

### Statistical- and functional analysis

Raw intensities were normalized by quantile normalization. Initial quality control investigated the effect of scanning date and array intensities. Further data analysis was done using OmniViz version 5.0 (Biowisdom), Partek, and R program. Minimum thresholds were set at 30. Scanning data effects on expression values were removed using Partek's software package.

As hypothesis 1 states that AKT1 expression is altered in PBMCs of patients, we investigated AKT1 expression levels between patients and controls, using Students t-test (SPSS 16.0 statistical software). Schizophrenia patients frequently abuse cannabis. This may influence AKT expression. As the control group is not matched to the patients with respect to the use of cannabis, we performed subgroup analysis within the patients to explore the influence of cannabis use.

To identify transcripts that were differentially expressed between patients and controls without an a priori hypothesis we used analysis of variance (ANOVA) with Benjamini-Hochberg correction for multiple comparisons, as provided by Partek's software package, with False Discovery Rate (FDR) set to 0.05. We did not specify a minimal Fold-Change (FC) cut-off value, as it has been shown that alterations in gene expression in AKT−/− mice can be significant and informative, though with minimal FC [15].

For both the pilot and the main study functional relationships between genes which were differentially expressed between patients and controls were investigated using the Ingenuity Pathway Analysis software tool (IPA -Ingenuity Systems®; www.ingenuity.com). The IPA version of October 2006 and January 2009 were used for the pilot study and the main study respectively. For canonical pathways we considered a p-value<0.05 as significant.

Based on our hypothesis we specifically investigated the presence of genetic networks and canonical pathways in which AKT1 plays a role.

**Table 1** gives a flow-chart overview of the analyses performed, and in which tables the results of the analyses can be found.

**Table 1:** Flowchart description of the analyses.

Step:	Procedure:	Results can be found in:
**1**	**Micro-array gene expression analysis**	
	Quantile normalization of raw intensities	
	Expression threshold >30	
	Quality Control	
	2 patients removed because of outlying signal intensities	
	In final analysis: 41 patients and 29 controls	**Table 2**
**2**	**Test of hypothesis 1**	
	Investigate PBMC AKT1 expression (t-test) in patients vs controls	**Table 3** ** and ** **figure 1**
	Investigate AKT1 expression between patient subgroups (antipsychotic naïve/free vs medicated, nicotine vs non-nicotine, cannabis vs non-cannabis)	**Table 3** ** and ** **figure 1**
**3**	**Identify all transcripts differentially expressed between patients and controls**	
	Using the complete expressed set of transcripts:	
	Identify transcripts differentially expressed between patients and controls (ANOVA, Benjamini-Hochberg correction for multiple comparisons, FDR<0.05)	**supporting table S2**
**4**	Upload differentially expressed transcripts into Ingenuity Pathway Analyzer (IPA)	
**5**	Investigate general functional aspects of the set of differentially expressed transcripts	Text, subheading “**pathway analysis**”
**6**	**Test of hypothesis 2**	
	Investigate presence of the differentially expressed transcripts in metabolic and functional processes using ‘*canonical pathway*’ function of the IPA	**Table 4**
	Specifically investigate AKT1-related pathways	Table 4

Initially included: 46 patients and 30 controls.

Excluded from analysis: 3 patients and 1 control (2 patients: somatic disorders discovered after inclusion; 1 patient and 1 control: insufficient RNA obtained).

Used for analysis: 43 patients and 29 controls.

## Results

### Subjects

We initially included 46 patients, and 30 controls in the study. Of these, a total of 5 patients and 1 control were excluded: two patients were excluded because of somatic disorders which were identified after inclusion (one patient suffered from microcytic anemia, one other patient of nephrotic syndrome); 1 patient and 1 control were not included because too little RNA was obtained, and 2 more patients were excluded post-scanning because build-in quality controls indicated poor quality of these samples.

The mean ages of the remaining 41 patients (23.02 (SD 4.03)) and 29 controls (23.90 (SD 4.08) years) were not significantly different: p = 0.378. Eleven patients were free of antipsychotic medication for more than 2 weeks. See **table 2** for the complete patient characteristics.

**Table 2:** Patient characteristics.

		Patients (N = 41)	Controls (N = 29)
Smoking		yes: 26 (63.4%)	Yes: 5 (17,2%)
		no: 15 (36.6%)	No: 24 (82,8%)
Cannabis abuse		yes: 18 (43.9%)	Yes: 2 (6,9%)
		no: 23 (56.1%)	No: 27 (93,1%)
Duration of illness (DUI) (weeks) (N = 36)†		116.22 (SD 99.89) weeks	
		min-max: 1–250 weeks	
		median: 92.50 weeks	
**Ethnicity**			
	European (mostly Dutch descent)	20 (48.8%)	24 (82,6%)
	Surinamese/African descent	6 (14.6%)	1 (3,4%)
	Cape Verdean	1 (2.4%)	0 (0,0%)
	Surinamese/Hindustani	6 (14.6%)	1 (3,4%)
	Moroccan/North African	2 (4.9%)	0 (0,0%)
	Asian	1 (2.4%)	1 (3,4%)
	Mixed	3 (7.3%)	2 (6,9%)
	Could not be reliably assessed	2 (4.9%)	
**Antipsychotics used at study entry**			
	naive (mean age naive: 5.0 (SD 4.7) years)	6 (14.6%)	
	free>2 weeks, not naive (mean age free>2 weeks: 25.2 (SD 3.9) years)	5 (12.2%)	
	total (free>2 weeks or naïve)	11 (26.8%)	
	haloperidol	9 (22.0%)	
	risperidone	1 (2.4%)	
	olanzapine	4 (9.8%)	
	quetiapine	1 (2.4%)	
	clozapine	15 (36.6%)	
**PANSS scores**			
	total score	77.7 (SD 14.4)	
	positive subcale	18.5 (SD 6.6)	
	negative subscale	22.0 (SD 5.8)	
	general psychopathology	37.3 (SD 8.0)	

† of 5 patients DUI could not be reliably assessed.

### AKT1 expression between patients and controls

We found decreased levels of expression of AKT1 (log-transformed relative intensity values) in the patients (518.58 (SD 61.46) versus the controls (580.48 (SD 57.91)); t = −4.250, df = 68, p<0.001; 95% CI −90.97; −32.84. The effect size of the difference in the means is large (eta squared: 0.21).

Expression levels of AKT1 between acutely admitted (florid psychotic) and remitted patients were not significantly different (528.92 (SD 67.34 versus 508.73 (SD 55.13)), p = 0.299.

**Figure 1:**
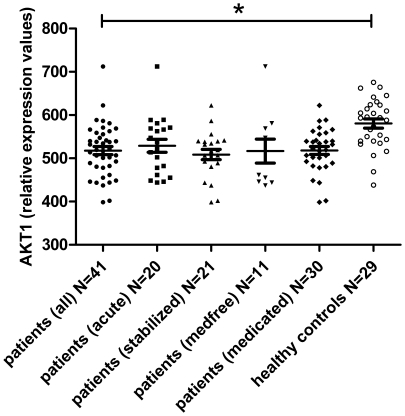
Scatter plots of PBMC AKT1 expression levels between patients, patient subgroups, and controls. Cross-lines show mean and standard error of the mean. * p<0.001 (independent samples t-test).

Expression levels of AKT1 in antipsychotic-free patients (493.33 (SD 55.09) were not significantly different from the medicated patients (526.89 (SD 63.06); t = 1.449, df = 39, p = 0.155; 95% CI −12.25; 74.21, and still significantly different from the controls (N = 29; 580.48 (SD 57.91); t = −4.220, df = 38, p<0.001; 95% CI −125.18; −43.96). Also, AKT1 expression in antipsychotic-naïve patients (N = 6) was not significantly different as compared to medicated patients (N = 30) (p = 0.114). **See** **figure 1**.

Indeed, if any, AKT1 expression levels tended to be higher in the medicated patients.

Also, AKT1 expression in patients using nicotine (N = 26; 530.45 (SD 58.31)) was not significantly different from those who did not smoke (N = 15; 498.00 (SD 63.29): p = 0.104. Neither was AKT1 expression in patients using cannabis significantly different as compared to patients not using cannabis (p = 0.691). Also in these cases, if any, patients with nicotine or cannabis abuse tended towards higher level of AKT1 expression. **Table 3** gives a complete overview of AKT1 expression values in patient subgroups.

**Table 3:** Overview of mean levels of PBMC AKT1 expression levels (log-transformed relative intensity values) between patients and controls and between patient subgroups.

	Controls	Patients (all)	Patients (acute) vs patients (stabilized)	Patients (antipsychotic naïve)	Patients (antipsychotic free>2 weeks)	Patients (medicated)	Patients using nicotine vs non-nicotine	Patients using cannabis vs non-cannabis
	(N = 29)	(N = 41)	(N = 20, N = 21)	(N = 6)	(N = 11)	(N = 30)	(N = 26, N = 15)	(N = 18, N = 23)
AKT1 expression(mean ± sd)	580.48±57.91	518.58±61.46*	528.92±67.34 vs 508.73±55.13†	481.81±41.23‡	493.33±55.09‡	526.72±62.01*	530.45±58.31 vs 98.00±63.29††	522.97±65.88 vs 515.14±59.05‡‡

* p<0.001 as compared to the controls (t-test).

† non-significant difference between acutely admitted patients vs stabilized patients.

†† non-significant difference between nicotine and non-nicotine.

‡ non-significant difference as compared to medicated patients.

‡‡ non-significant difference between cannabis abuse vs non-cannabis abuse.

AKT1 expression did not correlate with age (r: −0.046, p = 0.704), duration of iIlness (r: 0.242, p = 0.155), PANSS positive- (r: 0.120, p = 0.501), or PANSS negative subscales (r: 0.129, p = 0.466).

### AKT isoforms and GSK-3A/3B expression

The AKT isoforms, AKT2 and AKT3, both showed a trend toward decreased expression in the patients versus the controls. AKT2: 255.83 (SD 28.32) versus 269.21 (SD 30.82), p = 0.07; AKT3: 240.88 (SD 36.60) versus 260.59 (SD 44.15), p = 0.06. The important downstream targets of AKT, GSK-3A (p = 0.20) and GSK-3B (p = 0.97) did not show altered levels of expression

### Significance analysis of the complete set of genes

ANOVA showed 1224 transcripts that were significantly deregulated between patients and controls, after Benjamini-Hochberg correction for multiple comparisons (FDR = 0.05). See **supporting table S2** for the complete list of differentially expressed genes. Of the 1224 deregulated transcripts, 272 were upregulated, 952 were downregulated.

AKT1 is among these 1224 transcripts, so AKT1 remains differentially expressed between patients and controls also after Benjamini-Hochberg correction (p = 0.0005).

### Pathway analyses (see also table 1: flowchart of the analyses)

Next, we investigated the involvement of the 1224 initially identified probe sets in metabolic and signaling pathways using the ‘canonical pathway’ function of the IPA. This identified 35 canonical pathways in which more transcripts from the 1224 probe dataset participated than expected by chance (p<0.01), and 13 pathways using a more stringent criterion (p<0.001). Of the 35 deregulated pathways, AKT1 is involved in 20 pathways (57%). Using the more stringent criterion of p<0.001 as significance threshold, AKT1 is involved in 6 of the 13 deregulated pathways (46%). **Table 4** shows the deregulated canonical pathways, its significance, and in which pathways AKT1 participates.

**Table 4:** Canonical pathways deregulated between patients and controls.

Canonical pathway	Significance (IPA)
**Natural killer cell signaling**	**0.00000036**
SAP/JNK Signaling	0.00000283
Leucocyte Extravasation Signaling	0.00000304
T Cell Receptor Signaling	0.0000178
**Huntington's disease Signaling**	**0.0000334**
Inositol Phosphate Metabolism	0.000105
Cell Cycle: G1/S Checkpoint Regulation	0.000114
**Integrin Signaling**	**0.00019**
**Fc Epsilon RI Signaling**	**0.00021**
**B Cell Receptor Signaling**	**0.00025**
**Insulin Receptor Signaling**	**0.00054**
**JAK/Stat Signaling**	**0.001**
**p53 Signaling**	**0.001**
PDGF Signaling	0.001
**PTEN Signaling**	**0.001**
Apoptosis Signaling	0.002
Nicotinate and Nicotinamide Metabolism	0.002
**Axonal guidance Signaling**	**0.003**
**Ephrin receptor Signaling**	**0.004**
**Neurotrophin/Trk Signaling**	**0.004**
PPARα/RXRα activation	0.005
**NRF2-mediated oxidative stress response**	**0.008**
TGF-β Signaling	0.01
**Hypoxia Signaling in the Cardiovascular System**	**0.01**
**FGF Signaling**	**0.01**
**IL-2 Signaling**	**0.01**
Wnt/β-catenin Signaling	0.01
ERK/MAPK Signaling	0.01
**IGF-1 Signaling**	**0.01**
Xenobiotic metabolism Signaling	0.02
**IL-4 Signaling**	**0.03**
Aryl Hydrocarbon Receptor Signaling	0.03
**VEGF Signaling**	**0.03**
**Neuregulin Signaling**	**0.04**
Protein Ubiquitination Pathway	0.04

Significance values as supplied by the IPA. Pathways in bold are pathways in which AKT1 participates.

Further inspection shows that deregulated canonical pathways are related to immune processes, cell adhesion and neuronal guidance, neurotrophin signaling and (neural) growth factors, oxidative stress and glucose metabolism, and apoptosis and cell-cycle regulation.

## Discussion

In this study we show decreased expression of AKT1 in PBMC's of young, recent-onset, male schizophrenia patients. To our best knowledge this is the first confirmation of aberrant AKT1 expression in PBMC's of schizophrenia patients after the initial report by Emamian et al. [12] who investigated AKT1 protein levels in cultured lymphocyte-derived cell lines with SDS-PAGE and immunoblot analysis. We extend their findings by showing that AKT1 is also decreased in non-cultured PBMC's of schizophrenia patients, collected in a clinical setting, and that decreased expression is also observed in medication-free or -naive patients.

In addition, we demonstrate significantly deregulated canonical pathways in PBMC's of patients, in approximately 50% of which AKT1 is a key enzyme. We also show that deregulated canonical pathways tend to cluster in families that are related to immune processes, cell adhesion and neuronal guidance as well as apoptosis and cell-cycle regulation, neurotrophin signaling and (neural) growth factors, and oxidative stress and glucose metabolism.

AKT1 expression was decreased in both the acutely admitted florid psychotic patients, as well as in the remitted patients. Importantly, AKT1 expression was decreased in both medicated and unmedicated patients, indicating that decreased AKT expression is not a consequence of medication. Antipsychotic medication and nicotine or cannabis abuse were considered as possible confounders affecting the expression of AKT1. However, we could not identify significantly different levels of AKT1 expression between patients using antipsychotic medication and those who were antipsychotic free or –naïve, nor between drug abuse and non-abuse patients. If any, patients using medication or drugs tended towards higher AKT1 expression levels. So, antipsychotic medication or substance use probably cannot account for the decreased levels of PBMC AKT1 expression we found. Our study was, however, powered to find a main effect for AKT1 expression between patients and controls, but not for these subgroup analyses. So, our findings of decreased AKT1 expression in antipsychotic-naïve patients, and in those with cannabis abuse, should be interpreted with caution.

We could not identify a correlation between AKT1 expression, and age, duration of illness, and clinical symptom severity (PANSS positive and negative subscales).

Taken together, our observations suggest that decreased PBMC AKT1 expression is a stable trait in recent onset, male schizophrenia patients.

The relationship between AKT1 and schizophrenia has been previously described. Our findings significantly extend the work done in association studies on AKT1 and schizophrenia by showing decreased gene expression in a naturalistically obtained patient sample. Since the initial report of deregulated AKT1 in schizophrenia [12], several case/control samples and family cohorts showed an association between schizophrenia and AKT1 genetic variants [16]–[19]. However, as usual in association studies in schizophrenia, a number of negative findings have also been reported [20]–[24](for an updated compilation al all studies; see http://www.schizophreniaforum.org/res/sczgene/geneoverview.asp?geneid=6). Also a number of post-mortem studies show evidence of a decrease in AKT1 expression in brains of individuals with schizophrenia [12], [19], [25].

AKT comprises three closely related isoforms AKT1, AKT2, and AKT3. Although the AKT isoforms AKT2 and AKT3 did not pass correction for multiple testing, an uncorrected t-test showed a trend towards decreased expression for both AKT2 (p = 0.07) and AKT3 (p = 0.06). This observation suggests that decreased expression may be present for all three AKT isoforms. The three AKT isoforms were long thought to play redundant and overlapping roles. Recent work however pointed to differential roles played by AKT1 and AKT2 in a variety of cellular processes [26]. AKT1 has predominantly been associated with cellular survival and apoptosis, whereas AKT2 seems to be associated with insulin signaling and glucose metabolism.

We did not find altered expression of the main downstream target of AKT, GSK-3, in concordance with previous reports [27], [28].

From a conceptual perspective, the involvement of AKT in schizophrenia is plausible. Generally, one of the major functions of AKT is to promote growth-factor mediated cell survival and to block programmed cell death or apoptosis [29]–[31]. Altered apoptotic processes have repeatedly been proposed to underlie schizophrenia, and are part of the influential so-called ‘neurodevelopmental model of schizophrenia’ [32]–[34]. AKT is also a key signaling enzyme downstream of dopamine D2 receptors [35]–[37]. Aberrant dopaminergic neuromodulation is one of the most well-established findings in schizophrenia and is thought to underlie psychotic phenomena as well as cognitive disorders present in schizophrenia (i.e. see Kapur [38]).

Although the effect size of the difference in mean AKT1 values between patients and controls is large by statistical standards, the clinical utility of our finding is limited. The distribution of the data (see figure 1) clearly shows overlap between patients and controls. This may be due to biological heterogeneity underlying the schizophrenia syndrome. The modern conceptualization of the schizophrenia syndrome is that of a syndrome that originates from multiple interwoven neurodevelopmental processes [39], in interaction with environmental influences such as cannabis abuse or living in an urban environment [40]. So, outlying values of AKT1, as well as considerable overlap of values between patients and controls, may be due to this heterogeneity.

Besides AKT1, we found a total of 1224 transcripts deregulated between patients and controls in the main study. Of these, the down-regulated transcripts greatly outnumber the up-regulated ones, in an approximately 4∶1 ratio. This finding is in line with previous reports that (post-mortem) gene expression changes in subjects with schizophrenia are predominantly characterized by transcript reductions, rather than increases [41]–[43]. The magnitude of change however, although significantly altered, is limited for the vast majority of transcripts, pointing towards only subtle alterations in expression status. As PBMCs in schizophrenia do not show overt pathology, subtle alterations in gene-expression are what might be expected.

The results of the pathway analyses we performed show aberrantly regulated processes in patients to be related to a number of biological themes: immune processes, cell adhesion and neuronal guidance, neurotrophins and (neural) growth factors, oxidative stress and glucose metabolism, and apoptosis and cell-cycle regulation (see table 4). A considerable number of significantly changed canonical pathways point towards the presence of an altered immune status in patients (**pathways which include AKT1 are in italics**: *Natural killer cell signaling*, Leucocyte Extravasation Signaling, T Cell Receptor Signaling, *Fc Epsilon RI Signaling*, *B Cell Receptor Signaling*, *IL-2 Signaling*, Xenobiotic metabolism Signaling, and *IL-4 Signaling*; see also table 3). The presence of an immunological component in schizophrenia has been hypothesized, based on several convergent findings [42], [44]–[48]. However, we want to point out that that the finding of immune perturbations could well be an epiphenomenon of the specific tissue under investigation.

The presence of deregulation of *Integrin Signaling*, *Axonal guidance Signaling*, *Ephrin receptor Signaling*, and *Neuregulin Signaling* (table 3), all of which are associated with neuronal outgrowth, cellular patterning during development, and cell adhesion, combined with the presence of deregulated, *p53 Signaling*, *PTEN Signaling*, apoptosis Signaling)(see table 3) may indeed point to the presence of aberrant neurodevelopmental processes. We also find the *neurotrophin/Trk signaling* pathway deregulated, as well as some other growth factor and cytokine related pathways (PDGF Signaling, TGF-β Signaling, *FGF Signaling*, *IGF-1 Signaling*, *VEGF Signaling*)(table 3). Neurotrophins are a specific subset of growth factors, most notably among them Brain-Derived Neurotrophic Factor (BDNF). There is an emerging body of converging evidence that points to a relation between schizophrenia and disrupted levels of BDNF, both in the central nervous system and in peripheral blood (see [49], [50] for a review). The biological responses of neurotrophins after activating the neurotrophin/trk pathway include among others proliferation and survival, and axonal and dendritic growth and remodeling.

Finally, we find several deregulated canonical pathways which may point towards the presence of altered insulin signaling and oxidative stress (*Inositol Phosphate Metabolism*, *Insulin Receptor Signaling*, *NRF2-mediated oxidative stress response*, *IGF-1 Signaling*, and *Hypoxia Signaling in the Cardiovascular System*)(see table 3), pointing towards alterations in glucose metabolism in schizophrenia, as have been demonstrated to exist not only secondary to the use of antipsychotics, but also in antipsychotic-naïve patients [25], [51]–[53].

There are several strengths and limitations to our study. We consider a specific strength the deferred consent procedure we used, which enabled us to include severely disturbed, antipsychotic free, or even –naïve patients. Furthermore, we rigorously eliminated confounders during the laboratory procedures, such as blood sampling by various staff members, inclusion at several sites with associated multiple transport, storage and/or laboratory procedures, and bench work by multiple scientists.

There are however, limitations of our study. First, we studied PBMCs and the findings may not truly reflect changes in the brain. Second, the majority of patients were on antipsychotic medication. Several reports show that antipsychotics (haloperidol as well as olanzapine and clozapine) influence AKT. However, the majority of reports show that antipsychotics do not influence *expression* levels of AKT, but only increase the phosphorylation status of AKT, thus increasing AKT1 activity without influencing its expression levels. This issue is extensively discussed in a recent overview by Freyberg et al. [54]. Our results show that AKT1 expression is also decreased in the antipsychotic-free or -naïve patients, in line with these findings.

We did not investigate patients with bipolar disorder or major depression. Recent insights based on family and genetic studies have suggested that especially bipolar disorder and schizophrenia have a number of factors in common [55]. Data on the relationship between AKT1 and bipolar disorder is scarce however. Thiselton et al [18] measured mRNA levels of both AKT1 and AKT2 isoforms in post-mortem brain tissue and found a significant decrease in schizophrenia (AKT1) and in bipolar disorder (AKT2). It may be fruitful to also investigate AKT levels in peripheral tissue in bipolar disorder.

In conclusion, we show decreased PBMC gene expression of AKT1 in schizophrenia patients, with a considerable effect size, which allowed detection in two relative small cohorts. A large number of AKT-related functions, processes and canonical pathways are deregulated between patients and controls. A majority of these have previously been associated with schizophrenia. Specific attention merits the presence of an altered immune status in the patients as well as a deregulated status of genes associated with cell growth, cell guidance, cell adhesion, (developmental) spatial patterning, and energy metabolism. (Cultured) PBMCs may be useful tissue in which to investigate aspects of the molecular biology of schizophrenia.

## Supporting Information

Table S1 **Data pilot study. Pilot data methods:** *Subject selection, inclusion criteria, laboratory procedures* as described for the main study *criterion for deregulated genes:* t-test p<0.05 (no correction for multiple comparisons was applied as this was a hypothesis generating study) ánd Fold Change >1.2.
(DOC)

Table S2 **List of deregulated genes.** Transcripts differentially expressed between patients and controls; Benjamini-Hochberg correction for multiple comparisons, False Discovery Rate (FDR) set to 0.05 (see also main text).
(XLSX)
