# Supplementary material for: Marked Reduction of AKT1 Expression and Deregulation of AKT1-Associated Pathways in Peripheral Blood Mononuclear Cells of Schizophrenia Patients
Source: PLoS One. 2012 Feb 29;7(2):e32618. doi: 10.1371/journal.pone.0032618 (PMC3290567; doi:10.1371/journal.pone.0032618)
Supplement: Table S1 — Data pilot study. Pilot data methods: Subject selection, inclusion criteria, laboratory procedures as described for the main study criterion for deregulated genes: t-test p<0.05 (no correction for multiple comparisons was applied as this was a hypothesis generating study) ánd Fold Change >1.2. (DOC) [file pone.0032618.s001.doc]

**SUPPORTING INFORMATION Table S1:**

**Pilot study; subject characteristics:**

|  | **Patients**  **(N=8)** | **Controls**  **(N=8)** |
| --- | --- | --- |
| Mean age (±sd) | 24.0 (5.1) | 23.1 (1.2) p=.643 |
| Etnicity | Caucasian (N=7)  Asian (N=1) | Caucasian (N=7)  Asian (N=1) |
| Duration of untreated psychosis (weeks (±sd)) | 69.8 (67.5) weeks |  |
| Duration of illness  (weeks (±sd)) | 152.6 (84.9) weeks |  |
| Medication | Clozapine (N=6)  Risperidone (N=1)  Medication free > 2weeks (N=1) |  |
| Cumulative lifetime antipsychotic usage (haloperidol eq (±sd)) | 3463 (1835) mg |  |
| PANSS total (±sd) | 68.8 (9.6) |  |
| PANSS positive (±sd) | 10.3 (1.5) |  |
| PANSS negative (±sd) | 25.7 (6.2) |  |
| PANSS general (±sd) | 32.8 (4.1) |  |

**Pilot data; results 1:**

**most significantly disregulated focus network as identified by the IPA (version October 2006)**

| **id** | **genes** | **score** | **focus genes** | **top functions** |
| --- | --- | --- | --- | --- |
| 1 | AKT1, ARHGEF2, BCR, BIRC1, CALR, CARD11, CCL5, FBXO32, GAB3, GADD45B, GATA3, GNG2, GRB2, Hd (Huntington), HNRPL, IKBKB, KLF13, MAF, MALT1, MAP4K4, MAPK8, MAPK9, MARK4, NFATC2, NFATC3, OPTN, PAK1IP1, PPIF, SOS1, TAP2, THBS1, TLR2, TNFRSF10A, TNFRSF10B, ZAK | 56 | 35 | Immune Response, Cell-To-Cell Signaling and Interaction, Cellular Development |

NB The significance score 56 is the highest that that can be achieved in the IPA

**Pilot data; results 2:**

**visualization of the most significantly deregulated focus network (IPA score 52).** The network shows functional relationships between genes.


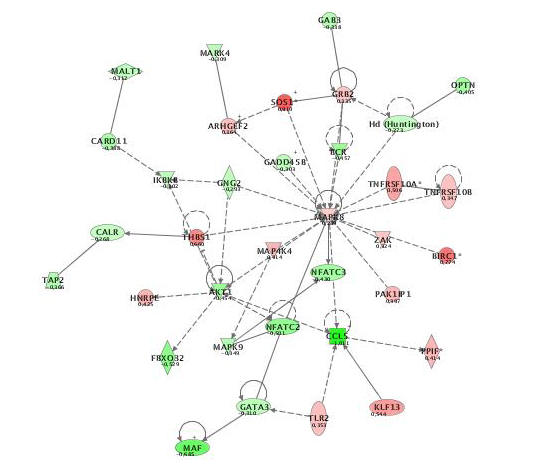


**Network annotation in terms of node shape:**

circle: other; hashed square: growth factor; hashed rectangle: ion channel; diamond: enzyme; oval: transcription factor; triangle facing down: kinase; and triangle facing up: phosphatase.

Edge types: line with arrow, acts on and line without arrow, binds to.

*: multiple probe sets in dataset for this gene.

The figures within within the nodes represent log2 fold change in expression in the patients vs the controls; negative values (green nodes) imply decreased expression in the patients and positive values (red nodes) imply increased expression in the patients.

Gene abbreviations:

AKT1: v-akt murine thymoma viral oncogene homolog 1- ARHGEF2: rho/rac guanine nucleotide exchange factor (GEF) 2 - BCR: breakpoint cluster region - BIRC1: baculoviral IAP repeat-containing 1 - CALR: calreticulin - CARD11: caspase recruitment domain family, member 11 - CCL5: chemokine (C-C motif) ligand 5 - FBXO32: F-box protein 32 - GAB3: GRB2-associated binding protein 3 - GADD45B: growth arrest and DNA-damage-inducible, beta - GATA3: GATA binding protein 3 - GNG2: Guanine nucleotide binding protein (G protein), gamma 2 - GRB2: growth factor receptor-bound protein 2- HD: huntingtin (Huntington disease) - HNRPL: heterogeneous nuclear ribonucleoprotein L - IKBKB: inhibitor of kappa light polypeptide gene enhancer in B-cells, kinase beta - KLF13: Kruppel-like factor 13 - MAF: v-maf musculoaponeurotic fibrosarcoma oncogene homolog (avian) - MALT1: mucosa associated lymphoid tissue lymphoma translocation gene 1 - MAP4K4: mitogen-activated protein kinase kinase kinase kinase 4 - MAPK8: mitogen-activated protein kinase 8 - MAPK9: Mitogen-activated protein kinase 9 - MARK4: MAP/microtubule affinity-regulating kinase 4 - NFATC2: Nuclear factor of activated T-cells, cytoplasmic, calcineurin-dependent 2- NFATC3: nuclear factor of activated T-cells, cytoplasmic, calcineurin-dependent 3 - OPTN: optineurin - PAK1IP1: PAK1 interacting protein 1 - PPIF: peptidylprolyl isomerase F (cyclophilin F) - SOS1: son of sevenless homolog 1 (Drosophila) - TAP2: transporter 2, ATP-binding cassette, sub-family B (MDR/TAP) - THBS1: thrombospondin 1 - TLR2: toll-like receptor 2 - TNFRSF10A: tumor necrosis factor receptor superfamily, member 10a: TNFRSF10B: tumor necrosis factor receptor superfamily, member 10b - ZAK: sterile alpha motif and leucine zipper containing kinase AZK

**Pilot data; results 3:**

**deregulated canonical pathways (P<0.001) as identified by the IPA. Significances as provided by the IPA.**

| **canonical pathway** | **Significance** |
| --- | --- |
| Interferon signaling | 0.000005 |
| EGF signaling | 0.00006 |
| IL-6 signaling | 0.00008 |
| PI3K/Akt signaling | 0.00001 |
| B cell receptor signaling | 0.0002 |
| IL-4 signaling | 0.0004 |
| SAP/JNK signaling | 0.0006 |
| IL-2 signaling | 0.0006 |
| T cell receptor signaling | 0.0007 |

**Pilot data; results 4:**

**boxplot diagram of AKT1 expression between patients and controls**

(df=14; t=3.051; p<0.009)
